# Participants’ and Nurses’ Experiences With a Digital Intervention for Patients With Depressive Symptoms and Comorbid Hypertension or Diabetes in Peru: Qualitative Post–Randomized Controlled Trial Study

**DOI:** 10.2196/35486

**Published:** 2022-09-15

**Authors:** Mauricio Toyama, Victoria Cavero, Ricardo Araya, Paulo Rossi Menezes, David C Mohr, J Jaime Miranda, Francisco Diez-Canseco

**Affiliations:** 1 CRONICAS Center of Excellence in Chronic Diseases Universidad Peruana Cayetano Heredia Lima Peru; 2 Centre for Global Mental Health, Institute of Psychiatry, Psychology, and Neuroscience King's College London London United Kingdom; 3 Population Mental Health Research Centre Universidade de São Paulo São Paulo Brazil; 4 Department of Preventive Medicine, Faculdade de Medicina Universidade de São Paulo São Paulo Brazil; 5 Center for Behavioral Intervention Technologies, Department of Preventive Medicine Feinberg School of Medicine Northwestern University Chicago, IL United States

**Keywords:** mobile intervention, depression, diabetes, hypertension, comorbidity, qualitative research, mobile phone

## Abstract

**Background:**

Depression is one of the most prevalent mental disorders and a leading cause of disability, disproportionately affecting specific groups, such as patients with noncommunicable diseases. Over the past decade, digital interventions have been developed to provide treatment for these patients. CONEMO (Emotional Control in Spanish) is an 18-session psychoeducational digital intervention delivered through a smartphone app and minimally supported by a nurse. CONEMO demonstrated effectiveness in reducing depressive symptoms through a randomized controlled trial (RCT) among patients with diabetes, hypertension, or both, in Lima, Peru. However, in addition to clinical outcomes, it is important to explore users’ experiences, satisfaction, and perceptions of usability and acceptability, which can affect their engagement with the intervention.

**Objective:**

This study aimed to explore the RCT participants’ experiences with CONEMO in Peru, complemented with information provided by the nurses who monitored them.

**Methods:**

In 2018, semistructured interviews were conducted with a sample of 29 (13.4%) patients from the 217 patients who participated in the CONEMO intervention in Peru and the 3 hired nurses who supported its delivery. Interviewees were selected at random based on their adherence to the digital intervention (0-5, 10-14, and 15-18 sessions completed), to include different points of view. Content analysis was conducted to analyze the interviews.

**Results:**

Participants’ mean age was 64.4 (SD 8.5) years, and 79% (23/29) of them were women. Most of the interviewed participants (21/29, 72%) stated that CONEMO fulfilled their expectations and identified positive changes in their physical and mental health after using it. Some of these improvements were related to their thoughts and feelings (eg, think differently, be more optimistic, and feel calmer), whereas others were related to their routines (eg, go out more and improve health-related habits). Most participants (19/29, 66%) reported not having previous experience with using smartphones, and despite experiencing some initial difficulties, they managed to use CONEMO. The most valued features of the app were the videos and activities proposed for the participant to perform. Most participants (27/29, 93%) had a good opinion about the study nurses and reported feeling supported by them. A few participants provided suggestions to improve the intervention, which included adding more videos, making the sessions’ text simple, extending the length of the intervention, and improving the training session with long explanations.

**Conclusions:**

The findings of this qualitative study provide further support and contextualize the positive results found in the CONEMO RCT, including insights into the key features that made the intervention effective and engaging. The participants’ experience with the smartphone and CONEMO app reveal that it is feasible to be used by people with little knowledge of technology. In addition, the study identified suggestions to improve the CONEMO intervention for its future scale-up.

**Trial Registration:**

ClinicalTrials.gov NCT03026426; https://clinicaltrials.gov/ct2/show/NCT03026426

## Introduction

### Background

Depression is one of the most prevalent mental disorders worldwide, affecting >322 million people, and it is the leading cause of global disability [1]. Depression is more prevalent in specific groups, including patients with chronic noncommunicable diseases, such as diabetes and hypertension, negatively affecting their treatment adherence and health outcomes [2]. Despite this, there is still a significant treatment gap for mental disorders, particularly in Latin America [3].

There have been increasing efforts to improve this situation over the last decade, including digital and internet-based interventions for mental disorders [4], which have proven to be effective in reducing depressive symptoms [5]. However, they also come with their own set of challenges, particularly for older populations, who are more likely to be affected by comorbid depression and diabetes or hypertension. Issues such as physical disabilities and low technology literacy negatively affect their experience with and adherence to digital interventions. In contrast, positive experiences and perceived benefits improve engagement and adherence to them [6-9].

Randomized controlled trials (RCTs) are the most rigorous method to assess the effectiveness of an intervention [10], and several digital and internet-based interventions have proven to be effective in treating depression [11,12]. Although RCTs usually focus on the clinical outcomes, it is also important to explore the users’ experiences, as poor usability and low acceptability can lead to low engagement with the intervention and user errors and ultimately reduce the potential effectiveness of the digital intervention [13,14]. Consequently, assessing the users’ experiences can help researchers to better understand the key features of engagement with a digital intervention, what needs improvement, and which benefits are more relevant, to further inform changes to improve its design. The assessment of users’ experience with digital interventions is complex but usually focuses on 2 key components: usability and acceptability [13-16], and they are usually explored through qualitative methods [16].

This paper presents the findings of a qualitative study conducted by the Latin American Treatment and Innovation Network in Mental Health [17] as part of the evaluation of the CONEMO (Emotional Control in Spanish) RCT. CONEMO is a 6-week low-intensity psychoeducational digital intervention designed to reduce depressive symptoms among people with diabetes, hypertension, or both, delivered through a smartphone app and minimally supported by a nurse.

### The CONEMO RCT

The aim of the RCT was to assess the effectiveness of the CONEMO intervention in reducing depressive symptoms among individuals with diabetes, hypertension, or both, attending public health care facilities in Lima, Peru, and São Paulo, Brazil [17]. The RCT was preceded by pilot studies in both cities, which showed that the trial was feasible to be conducted in public services and presented promising results for the intervention, specifically, a trend in the reduction of depressive symptoms and improvements in disability levels [18].

The RCT results showed that the intervention was effective in reducing the baseline Patient Health Questionnaire-9 (PHQ-9) score by at least 50% at the 3-month follow-up compared with the enhanced usual care group [17]. In addition, there were improvements in disability, quality of life, and activity levels in the intervention group at 3 months. At the 6-month follow-up, only the improvement in activity levels was statistically significant [17].

### Objectives

This qualitative study aimed to explore the RCT participants’ experience with CONEMO in Peru, complemented with information provided by the 3 nurses who monitored them. Specifically, the study aimed to provide insights into (1) the participants’ satisfaction and acceptability of CONEMO, (2) the perceived benefits of its use, (3) their experience with the study nurses, (4) their experience with the usability of the smartphone and app, (5) the problems they encountered with technology, and (6) their suggestions to improve the intervention.

## Methods

### Design

This was a qualitative study, conducted after the 6-month follow-up assessment of the participants of the CONEMO RCT in Lima, Peru (ClinicalTrials.gov NCT03026426).

### Description of the CONEMO Implementation in Peru

**Figure 1:**
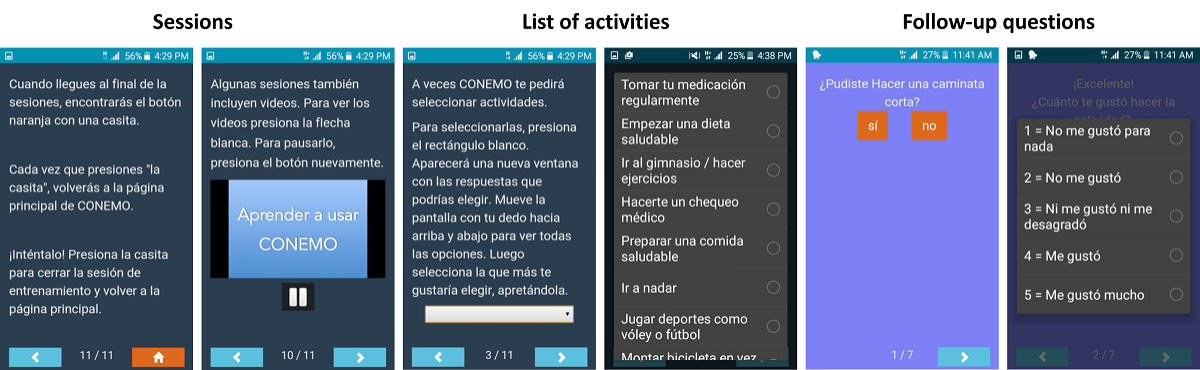
CONEMO app interface.

In Peru, 217 participants were randomly assigned to the digital intervention arm. They received a loaned smartphone with the app installed. The app had a basic interface, which allowed reading the latest session and checking previous completed sessions, and a *request help* button to use in case they encountered difficulties while using the app. The sessions consisted of texts, and some had videos in which a psychologist talked to the participants about the topic of the session based on the principles of behavioral activation [19]. Participants received 3 sessions per week for 6 weeks, completing up to 18 sessions in total. The sessions focused on promoting pleasant activities, healthy activities, and tasks (Figure 1). At the end of each session, participants were presented with a list of activities that they could aim to complete until the next session. At the beginning of the next session, the app presented follow-up questions asking the participants if they had completed the previously selected activity and how they felt about it. If the participant had not completed the activity, they received a message within the app, motivating them to complete it. Participants with high PHQ-9 score received a recommendation to seek specialized mental health care in their health facilities at inclusion in the RCT.

The intervention was supported by 3 nurses, hired full time for the project, who conducted an in-person 1-hour training with the participants on the use of the smartphone and app, monitored their adherence to the sessions, and provided technical support, if necessary. Participants received a manual describing the use of the app. For monitoring, nurses made 2 mandatory monitoring phone calls in the initial 3 weeks and additional phone calls if the participants had low adherence (defined as not completing 2 consecutive sessions) or requested help. To monitor their assigned participants, nurses used a dashboard, which displayed the sessions completed by the participants and the help requests. At the end of the 6-week period, the nurses and participants had a final meeting in the health clinic to return the smartphone. During the RCT in Peru, each study nurse had between 72 and 74 participants assigned to them over a period of 9 months. The nurses’ activities are described in further detail in another publication [20].

### Setting and Informants

In Peru, the RCT was conducted in 3 public hospitals and 4 public primary health care centers located in Lima, the capital city. The RCT procedures and inclusion criteria are described in the main paper [17].

The information for the qualitative study was collected from two types of informants: (1) participants who received the CONEMO intervention and (2) nurses involved in the intervention delivery during the RCT. The participants were adults with diabetes, hypertension, or both and depressive symptoms as measured using PHQ-9 at the time of inclusion in the RCT (score ≥10, which indicates moderate depressive symptoms). For this qualitative study, 2 hospitals and 2 primary health care centers were selected, where 312 (72.2%) of the 432 RCT participants were recruited.

We aimed to interview between 24 and 36 participants (6-9 participants per facility) and all the study nurses (3/3, 100%). To ensure representation of participants across levels of engagement with the digital intervention, 3 groups were predefined according to their adherence to the digital intervention during the RCT: low (0-5 sessions completed), medium (10-14 sessions completed), and high adherence (15-18 sessions completed). On the basis of the number of participants in each of these adherence groups, a sample of potential interviewees proportional to the strata was selected. Owing to the high adherence of the participants to the intervention during the RCT (169/217, 77.9% completing all sessions), most participants (24/29, 83%) were from the 15-18–sessions category. The participants to be interviewed were selected at random by an independent statistician. Participants who were not able to be contacted were replaced by another participant from the same group, also selected at random. All the nurses (3/3, 100%) participated in the study.

### Data Collection Tools

Semistructured interviews were conducted to collect information. The interview guides were based on topics developed for a research project by the National Institute of Mental Health Collaborative Hubs [21] and locally adapted by the Latin American Treatment and Innovation Network in Mental Health research team to make them relevant to the participants and nurses’ experiences with the CONEMO intervention. The topics covered in the 2 interview guides are listed in Table 1.

**Table 1:** Topics included in the interview guides.

Informants	Topics
RCT^a^ participants	Expectations of their participation in the RCT Satisfaction with their participation Acceptability of the intervention Perceived benefits of the intervention Experience with usability of the smartphone and app Difficulties found while using the smartphone or app Relationship with the study nurse Experience with the training and monitoring Suggestions to improve the intervention
Nurses	Experience with participants’ training Relationship with the participants Supervision of participants

^a^RCT: randomized controlled trial.

### Procedures

Data were collected from September 2018 to December 2018, upon completion of the 6-month evaluation of the RCT. Both interviewers and participants were blinded to the results of the RCT throughout data collection and analysis. Potential informants were contacted via phone and invited to participate in the study. After providing informed consent, informants were interviewed face-to-face by the RCT’s fieldwork coordinator (MT; male) or fieldwork supervisor (VC; female), 2 bachelors in psychology, with experience in conducting in-depth interviews. The interviewers discussed the interview guides and standardized their procedures. Individual interviews were conducted in Spanish, at the health facility in which the participant was recruited for the RCT. Interviews with nurses were conducted at the research team’s offices. The duration of interviews was, on average, 52 (SD 19) minutes for RCT participants and 71 (SD 18) minutes for nurses. All interviews were audio recorded and transcribed verbatim.

### Data Analysis

All interviews were analyzed using NVivo (version 12; QSR International), and content analysis was conducted [22]. A coding book was developed for each type of informant in advance, based on the interview guides. In total, 2 researchers (MT and VC) conducted the coding process after a standardization process. During coding, emerging codes were discussed between the coders to decide if they were to be added to the coding book. After coding was completed, quotes and codes were summarized using a coding matrix [23].

### Ethical Considerations 

The protocol of the RCT, including this qualitative study, was approved by the National Institute of Mental Health Data and Safety Monitoring Board and locally by the institutional review board at the Universidad Peruana Cayetano Heredia (Constancia 345-16-16). Participation was voluntary and all the informants signed an informed consent form before the interview. All the research team members completed ethical training in good clinical practices and human participants’ research.

## Results

### Participants’ Characteristics

Overall, 32 semistructured interviews were conducted with 29 people, who participated in the CONEMO intervention during the trial, and the 3 hired nurses, who supported its delivery. Participant demographics are shown in Table 2, and nurse demographics are shown in Table 3.

**Table 2:** Participants’ demographics (n=29).

Characteristics		Value
Age (years), mean (SD)		64.4 (8.5)
**Sex, n (%)**		
	Male	6 (21)
	Female	23 (79)
**CONEMO^a^ sessions completed, n (%)**		
	0-5	2 (7)
	10-14	3 (10)
	18	24 (83)
**Recruitment location (health facility), n (%)**		
	1	8 (28)
	2	9 (31)
	3	6 (21)
	4	6 (21)

^a^CONEMO: Emotional Control in Spanish.

**Table 3:** Nurses’ demographics (n=3).

Characteristics	Value
Age (years), mean (SD)	33 (5.2)
Sex (female), n (%)	3 (100)
Working experience (years), mean (SD)	8 (1.7)

### Participants’ Expectations and Satisfaction With CONEMO

The participants’ main expectations of the CONEMO intervention were to receive help to “feel better,” learn how to cope with their emotions, be motivated to try new things, better organize their activities throughout the day, communicate more with their children, learn more about how to deal with their diabetes, hypertension, or both, or do more exercise.

Most of the interviewed participants (21/29, 72%) stated that CONEMO fulfilled their expectations, which included feeling better and trying new things. In contrast, a few participants mentioned that their expectations were partially met or not met at all. For example, one of them said that he expected something “deeper” and more useful, but the intervention was mainly asking how he was feeling and to do things he already did, such as exercise and eating healthy:

Interviewer: “Okay, and do you consider that the study met your expectations, the expectations that you had at the beginning when you decided to participate?”

Participant: “No, because everything they said there, to eat vegetables, to do exercise, I have been doing that before, so just normal [activities], did not helped me much.”

Male participant; aged 68 years; high adherence

When asked if they would recommend CONEMO to other people, most interviewees (20/29, 69%) said that they would, some even stated that they had recommended it to their relatives or friends. The main reasons they recommended it were because it had helped them to feel better or improved their health or because they believed it can help other people. Some participants mentioned specific aspects of CONEMO that will be beneficial to others, for example, learning how to feel better and take care of themselves, receiving step-by-step instructions and advice, monitoring and attention received from the nurse, and recommendations to see a psychologist or psychiatrist:

I would recommend [CONEMO] to patients like me, who sometimes lose our memory, we forget, I forget one thing or another. And then, it makes you remember, because it tells you: take out your pen, write, do this, it makes you remember, it gives you very nice advice, that’s why I would perhaps advise another patient, that this is beautiful, it’s beautiful. Also, as I said, at our age no one pays attention to us and there was a nurse and a cellphone that they gave us and they were aware of me.

Female participant 1; aged 59 years; high adherence

### Perceived Benefits of Using CONEMO

Most participants (26/29, 90%) reported perceiving changes in themselves after using CONEMO, which helped them to feel better. Some of these changes were related to their thoughts and feelings, whereas others were related to their routine and habits.

#### Changes in Thoughts and Feelings

Regarding their thoughts and feelings, many interviewees (11/29, 38%) mentioned that they began to think differently or became more optimistic, and a participant mentioned that she stopped thinking about dying:

I thought about...what am I in this world for, right? My role is over, my children are already grown up, they can be on their own, like, my life no longer [had purpose]. I didn’t try to take my life, but I have thought about that. For example: “Oh, God, take me,” things like that, but no, I haven’t tried it. And I felt that I was falling down, but not now. Now I don’t think about it (laughs). Now I’m thinking that I have to travel (laughs) and now I’m doing it, right? And I feel better.

Female participant 2; aged 59 years; high adherence

In my case, I was depressed, I said: “what will happen to my life?” I tried to improve, thanks to CONEMO, thanks to the nurses, to the videos, ...reading it and practicing it, I tried to improve, that’s why I’m fine here, I feel good, I no longer feel depressed as before.

Male participant; aged 53 years; high adherence

Some participants mentioned feeling calm and more peaceful (8/29, 28%), whereas others said they felt more confident, paid more attention to themselves, and felt that they were important to other people (7/29, 24%). Another change mentioned was their interaction with others, including being less moody, listening more to others, and arguing less (5/29, 17%):

[CONEMO] calmed me down a lot, I felt safe, I felt that I was not, how do you say, a piece of furniture that is no longer used, because I was convinced that nobody cared about me, that I only mattered when they needed me. And [CONEMO] made me come out of all those things, all those doubts, all that concern and it helped me a lot.

Female participant; aged 69 years; medium adherence

There were a lot of changes in me, more independence, more confidence in myself and there are several things that I have achieved with CONEMO.

Male participant; aged 71 years; high adherence

#### Changes in Routine and Habits

Similarly, when asked about changes to their routines or habits, one of the most common changes was going out more (12/29, 41%). Some participants described their previous routines as being isolated and always staying at home, either owing to lack of motivation or physical difficulties, but after using CONEMO, they felt motivated to go out more often:

I started doing sports to feel better, right? Because it is good to walk, not to stay locked in the house. Sometimes you have problems and just stay locked in, so it is better to go out for a walk, walk, walk, be distracted, not overwhelmed with problems.

Female participant; aged 57 years; high adherence

Other changes perceived by the participants were developing new hobbies, including reading, listening to music, sewing, and knitting (6/29, 21%). Some participants developed interest in technology, and after they returned the smartphone provided by the study, their relatives bought them one to continue using it (4/29, 14%). Some participants also mentioned visiting or spending time with friends and family more often (5/29, 17%):

I knew sewing a bit and like it, so I made patterns and started drawing and taking measures, and out of the three [patterns] I made two blouses that I liked, and I still use them now. They are for summers but I use them, so all of that made me change.

Female participant; aged 76 years; medium adherence

Another important change mentioned by some participants was improving their health-related habits, such as taking care of their diabetes, hypertension, or both and being more consistent with taking their medication or eating healthy (4/29, 14%). Other participants mentioned doing more exercise (8/29, 28%), which was also perceived as a distraction, with a participant reporting having lost 25 kg since using CONEMO:

It was positive, yes, because it helped me with its questions, it also helped me to be more responsible with myself, with my medicines because I also forgot, I forgot to use my insulin on time...it supported me in that, it helped me.

Female participant; aged 57 years; high adherence

### Participants’ Experience With the Intervention

#### Experience With the Study Nurses

The study nurses were in charge of training the participants on how to use the smartphone and CONEMO app and provided monitoring to solve technical difficulties. Approximately two-thirds of the participants (18/29, 62%) considered the training clear and sufficient to learn how to use the smartphone and CONEMO app. However, nurses mentioned that this session was long and that some participants felt nervous about using the smartphone.

A few participants said that the training session with the nurse was not long enough to learn how to use the CONEMO app. They also sought help from a relative, and a participant mentioned learning through trial and error:

It was not enough, it was only one appointment with her, but no, I think it was very little time...like I told the nurse, I am a little bit dumb about learning this stuff, I have to practice a lot, the nurse told me if anything happens to call her, to let her know or ask for help, but I did not ask her because I thought “If I broke it?” but I asked help from my granddaughter.

Female participant; aged 76 years; medium adherence

Some participants reported experiencing some difficulties after the training, feeling that they initially understood the nurse’s instructions and information received, but forgot them later (6/29, 21%). Some participants scheduled a second training session with the nurse, whereas others asked a relative for help:

When she taught me, it seemed easy, but when being back at home, I forgot it, I did not know how to use it or anything.

Female participant 1; aged 59 years; high adherence

Regarding nurses’ phone monitoring, most of the interviewed participants (27/29, 93%) had a positive opinion, with some highlighting aspects such as feeling supported by the nurse, feeling important, and feeling that someone was interested in them. Other participants mentioned that the calls were useful either to reinforce continuing completing the sessions or as reminders to complete them:

I liked it because it showed an interest in me, and that is what I wanted, because I like to be listened to, to be taken care of, to have interest in me, and I felt that way, that they were interested. And I liked to be called.

Female participant 3; aged 59 years; high adherence

I usually forgot [the session], but [the nurse] called me so I remembered.

Male participant; aged 53 years; high adherence

Nurses agreed with this, stating that most of the participants were satisfied with the monitoring calls and only a few were reluctant to answer the phone or busy with other responsibilities:

I had a patient who blocked my number, she knew it was my number, so with the clinical coordinator we had to call her from public phones or another number to schedule [the monitoring], and I also had two, three patients who did not answer the calls or cancelled the appointment because they had to attend meetings with their children.

Nurse; aged 30 years

#### Experience With the Smartphone and App

Most of the interviewed participants (19/29, 66%) reported not having previous experience with using smartphones. Overall, approximately one-third of the interviewed participants (11/29, 38%) reported some difficulties at the beginning in learning how to use the smartphone provided by the study. They required some time to become familiar with the technology, but, eventually, they learned to use it. Nurses confirmed this, especially in the case of older adults:

At the beginning it was [difficult] to use the smartphone, just that, everything else was okay...a little bit difficult because I did not understand it, but then, little by little, reading the manual, I learned it all and I used it.

Female participant; aged 64 years; high adherence

In contrast, a few participants had a more negative opinion about their experience with the smartphone and considered it to be very difficult to use:

I do not like it. I have one [smartphone] given to me by my son, and I prefer this [basic] phone.

Female participant; aged 60 years; high adherence

Regarding the CONEMO app, most participants (28/29, 97%) had a good opinion, with most of them highlighting features such as the videos (19/29, 66%) and list of suggested activities for the participant to engage in (15/29, 52%). The videos were described as “educational” and “helpful,” and were clear and understandable to most participants (19/29, 66%). The activities proposed in the app were perceived as an opportunity to try new things or, through the app’s follow-up questions, to remind them to perform some activities. The participants compared these suggestions with having someone who cares about them:

It was good because [when] I started [with the study], I used to be secluded at home, but [then] I read, and went to visit my cousin, my friend. I went out to the beach to sit for a while and distract myself, I liked a lot that [CONEMO] told us to do these things.

Female participant; aged 74 years; low adherence

Some participants also liked reading the contents of the sessions and advice provided (10/29, 34%) and considered the contents to be “motivational.” Finally, another aspect of the app highlighted by 7% (2/29) of the participants was the frequency of the sessions (ie, 3 sessions per week), which was considered to be appropriate to take the time to perform the activities selected in each session:

It was ideal because it wasn’t every day, it was Monday, Wednesday, Thursday, I got a notification and with that I had a chance to make some time when I finished what I was doing to read and apply it.

Female participant 3; aged 59 years; high adherence

#### Problems Found While Using the Smartphone and App

Approximately half of the interviewed participants (15/29, 52%) reported having experienced some difficulties either with using the smartphone or the app. Regarding smartphone, the most common difficulties were related to not remembering how to perform certain basic tasks, such as using the touch screen, turning the smartphone on or off, or connectivity issues. Nurses noted that a common issue among participants was not remembering how to unlock the phone. Nurses also mentioned that at the beginning, they had difficulties in understanding their patients’ descriptions of problems with the phone during the calls because their explanations were not very clear. However, as the nurses became more familiar with those problems, they could help patients solve them more quickly:

Some patients had difficulties handling the phone, so I gave them options, like steps, so they can enter the phone easily...Practicing with them, taking the time to explain step by step, repeating it again and again, in the end they did understand what it was about.

Nurse; aged 39 years

Similarly, the most common difficulty found while using the app was not remembering how to use it. In addition, 7% (2/29) of the participants mentioned that they had difficulties in understanding the content of the sessions.

When queried about who they asked for help when encountering these difficulties, the most common answer was a young relative, followed by the study nurse. Other participants mentioned that they solved the issue by themselves through trial and error, reviewing the manual provided by the research team, or trying to remember the information provided by the nurse:

You know who helped the most? My granddaughter...I told her “Teach me because you know” I saw how she used her phone, so I told her “Teach me” and she said to me: “look, these pictures, messages, look, my uncle sent you a message.” Through her I did [learn].

Female participant; aged 76 years; medium adherence

### Suggestions to Improve the Intervention

When asked about their suggestions to improve different aspects of the intervention, only a few participants provided feedback, mostly centered on the app and its use. The most common suggestion was to add more videos and make them long, as this was the favorite feature of the app. Overall, 7% (2/29) of the participants suggested making the text of the sessions simple, because sometimes they found it difficult to understand. Others said that it should include more information, particularly about activities or hobbies that older people can do.

Other suggestions mentioned less frequently were to make the app more user-friendly, because some participants had to ask for help; increase the frequency of the app sessions; include more information and questions in them; and increase the font size. Some other suggestions were focused on the intervention design, with a few participants wanting the intervention to last long and include in-person meetings among the participants or to be able to interact with other participants through chat.

Very few participants had suggestions to improve the nurses’ activities. Some participants suggested having more contact with the nurse, either with more calls or more meetings. Others suggested improving the training with long explanations. Finally, a participant suggested receiving in-person help from the nurse to perform the activities proposed by the CONEMO app.

## Discussion

### Principal Findings

This study aimed to explore the experience of participants when using a digital intervention (CONEMO) while conducting an RCT to test this intervention in Lima, Peru. CONEMO was considered as an acceptable intervention by users, with most participants (26/29, 90%), including those with low adherence to the intervention sessions, reporting significant benefits for their mental and physical health and improvements in their habits and behaviors. In addition, regarding the usability of the technological components of the intervention, participants felt comfortable using the app; they experienced only a few difficulties, especially at the beginning, which they were able to solve with help from relatives or nurses. Collecting the participants’ experiences and opinions allowed a better understanding of the most valued aspects of the intervention and how they may have contributed to their engagement with the app during the RCT. In addition, this qualitative study provides important lessons that can guide the design of future digital mental health interventions, considering the feedback received from the participants.

During the RCT in Peru, the adherence rates for the CONEMO sessions were very high, with 92% of the participants completing more than half of the sessions and 78% completing all the 18 sessions [17]. The interviewed participants in the qualitative study acknowledged their satisfaction with the intervention, with most of them (21/29, 72%) mentioning that CONEMO fulfilled their expectations and that they would recommend it to other people. Furthermore, the participants elaborated on the perceived benefits of using CONEMO on their mood, thoughts, and behaviors, which provides context to the positive RCT results [17].

CONEMO was developed under the principles of behavioral activation [19], and the sessions invited participants to engage in different activities. In the RCT, participants in the intervention arm significantly improved their disability outcomes (as measured by the World Health Organization Disability Assessment Schedule-II) and levels of activity (as measured by the Behavioral Activation for Depression Scale Short Form) [17]. The qualitative interviews confirmed that CONEMO motivated the participants to engage in the proposed behavioral activation activities and introduced significant changes to their daily routines. We collected clear examples of the types and variety of activities they performed to be more vital and improve their physical and mental health. The study findings support the use of digital interventions based on behavioral activation to treat patients with depressive symptoms. In addition, it is aligned with existing evidence regarding the use of digital interventions to introduce lifestyle changes, particularly for older adults [24].

It is important to consider that the intervention included interaction with the project nurses. Despite the nurses’ role being centered on providing technical support related to the smartphone and CONEMO app, the interviewed participants stated feeling supported by the nurse and mentioned that they felt someone was interested in them. The participants were also particularly impressed with the videos in which they were able to see another person talking to them. It can be speculated that the feeling of support described by the participants owing to the interactive components of the intervention could have contributed to their engagement with CONEMO and the improvement in their emotional well-being. This finding is particularly relevant because the intervention is aimed toward people with noncommunicable diseases, who are mostly older adults and at great risk of experiencing social isolation [25,26], as described by some of the interviewees. Interactivity between the users and digital interventions has been reported as an important feature with a positive impact on the engagement with the intervention, particularly in mental health apps [13,27,28]. In addition, feedback and support have been reported as motivators for older adults using digital health interventions [29]. Digital interventions targeting older populations can include peer support, through chat or calls, as a feature of the app, as suggested by some interviewed participants.

Usability is a key component that influences the adoption of apps, particularly for people with little experience and knowledge of technology, and can influence engagement and symptom reduction [15]. In the case of CONEMO, although most participants (19/29, 66%) lacked experience in using a smartphone and encountered initial difficulties in becoming familiar with its use, most of them (24/29, 83%) were able to complete the sessions successfully and without great difficulties. This result highlights CONEMO’s good usability and confirms that older adults with no previous experience with technology are able to engage with digital interventions.

Considering the participants’ positive feedback regarding acceptability and usability, CONEMO can be easily adapted and implemented within the health system for patients with other health conditions or multimorbidity. The positive feedback has been echoed by other stakeholders who were interviewed, who consider CONEMO to be feasible to implement within the local health system [30]. In the current context of the COVID-19 pandemic, the population had to adapt relatively quickly to the use of technology to communicate with other people, work, and study, among other activities. Similarly, health systems also had to adjust quickly and implement telemedicine procedures where there were none. Particularly, within the Peruvian health system, the COVID-19 pandemic forced the health system to swiftly develop and implement telemedicine services and protocols [31]. This scenario makes the health system more open to introducing digital and technology-based interventions and provides an opportunity for CONEMO to be implemented on a wide scale. In addition, there is an ongoing mental health reform aimed to strengthen the provision of mental health services within the public health system [32,33]. CONEMO can be implemented in community mental health centers, which are increasing in number each year in Peru, and the study nurses’ role can be easily adopted by the available staff or other people extensively available in low-income and middle-income settings, such as community health workers, can be included.

Despite the success and positive feedback received from the participants, there are some improvements that can be made to the design of CONEMO for future interventions or deployment, and the interviews provided a few suggestions.

First, they suggested including more videos that were rated very favorably by the participants during the pilot study [18]. The similarity in the findings shows that the CONEMO videos are still highly valued by the participants and adding more will increase the satisfaction and engagement with the intervention.

Second, a few participants suggested reviewing the contents of the sessions to make them easy to understand and including further information on the types of activities they can perform and how to perform them. As a next step, it will be useful to conduct a validation process of the sessions’ content with a wide range of participants to make the sessions understandable and inclusive for all.

Finally, there is a perception among some participants that the training session for participants at the beginning of the intervention was not sufficient to fully understand how to use the smartphone and app, and a few participants suggested increasing the time for this activity. The revised training session can include an extra session to resolve doubts and allow time for more practice to foster familiarity with the device. In addition, the training should involve, when possible, a participant’s relative, as they were the main resource to solve the technical difficulties faced, and not the study nurses, as intended initially. Furthermore, as videos were a highly valued feature of the intervention, the training can also include videos to reinforce the content provided during the in-person training. Videos stored in an archive can be accessed at any time by participants or their relatives. These improvements will not only provide sufficient sources to solve technical difficulties but also optimize the use of the scarce human resources within the health systems of low-income and middle-income settings.

### Strengths and Limitations

The participant sample included people with different levels of adherence to the intervention and from different health facilities. In addition, we included study nurses to complement the information provided by the participants and compare their points of view. Given the high adherence to the intervention by most participants (200/217, 92.2% completed at least 9 sessions and 169/217, 77.9% completed all the sessions), it was difficult to include people with very low adherence. Another strength of this study is that everyone (ie, participants and evaluators) was blinded to the results of the trial, thus providing a less biased view of the intervention. A limitation of the study is the time at which the interviews were conducted. They were conducted approximately 4.5 months after the end of the 6-week intervention, and it is possible that their recollection of experiences may not be as accurate as one would expect when questions are asked immediately after completion of the intervention. It was not possible to conduct these interviews close to that time because the primary outcomes needed to be assessed 3 months after completion of treatment. However, the trained interviewers were instructed to ask additional questions and explore when the answers were very superficial or vague, and the data collected were sufficiently rich to capture the experiences of the participants.

### Conclusions

The findings of this qualitative study not only support but also enrich and complement the results found in the CONEMO RCT regarding improvements in the participants’ mental and physical health and the positive changes in their habits and daily behaviors. The participants’ experiences with the smartphone and CONEMO app showed that it is not only feasible to be used by people with little knowledge of technology but also, with minor adaptations, can be implemented at a large scale. In addition, the study identified suggestions to continue improving the CONEMO intervention and provided insights into the key features that made the intervention engaging and effective. Furthermore, these results provide further evidence that it is possible to implement digital mental health interventions with high rates of acceptability and adherence in low-income and middle-income countries.

## Abbreviations

CONEMO: Control Emotional in Spanish
PHQ-9: Patient Health Questionnaire-9
RCT: randomized controlled trial
